# The Impact of Genome Triplication on Tandem Gene Evolution in *Brassica rapa*

**DOI:** 10.3389/fpls.2012.00261

**Published:** 2012-11-29

**Authors:** Lu Fang, Feng Cheng, Jian Wu, Xiaowu Wang

**Affiliations:** ^1^Institute of Vegetables and Flowers, Chinese Academy of Agricultural SciencesBeijing, China

**Keywords:** whole genome duplication, tandem duplication, tandem gene evolution, *Brassica rapa*, *Arabidopsis thaliana*, *Arabidopsis lyrata*, *Thellungiella parvula*

## Abstract

Whole genome duplication (WGD) and tandem duplication (TD) are both important modes of gene expansion. However, how WGD influences tandemly duplicated genes is not well studied. We used *Brassica rapa*, which has undergone an additional genome triplication (WGT) and shares a common ancestor with *Arabidopsis thaliana*, *Arabidopsis lyrata*, and *Thellungiella parvula*, to investigate the impact of genome triplication on tandem gene evolution. We identified 2,137, 1,569, 1,751, and 1,135 tandem gene arrays in *B. rapa*, *A. thaliana*, *A. lyrata*, and *T. parvula* respectively. Among them, 414 conserved tandem arrays are shared by the three species without WGT, which were also considered as existing in the diploid ancestor of *B. rapa*. Thus, after genome triplication, *B. rapa* should have 1,242 tandem arrays according to the 414 conserved tandems. Here, we found 400 out of the 414 tandems had at least one syntenic ortholog in the genome of *B. rapa*. Furthermore, 294 out of the 400 shared syntenic orthologs maintain tandem arrays (more than one gene for each syntenic hit) in *B. rapa*. For the 294 tandem arrays, we obtained 426 copies of syntenic paralogous tandems in the triplicated genome of *B. rapa*. In this study, we demonstrated that tandem arrays in *B. rapa* were dramatically fractionated after WGT when compared either to non-tandem genes in the *B. rapa* genome or to the tandem arrays in closely related species that have not experienced a recent whole genome polyploidization event.

## Introduction

Gene copy number can be expanded through many ways, including whole genome duplication (WGD), tandem duplication (TD), segmental duplication, and gene transposition duplication. Among these four kinds of duplications, WGD played an important role in the evolution of eukaryotes and was well documented in many sequenced genomes (Semon and Wolfe, 2007; Edger and Pires, 2009). It has been demonstrated that most eudicot plants originated from an ancient hexaploid ancestor, followed by lineage-specific tetraploidizations in many taxas: one in *Populus* (Tuskan et al., 2006), two in *Arabidopsis* (Simillion et al., 2002; Blanc et al., 2003; Bowers et al., 2003), one in legumes (Cannon et al., 2006), three in *Brassica* (Wang et al., 2011), but none in *Vitis* (Jaillon et al., 2007), or papaya (Ming et al., 2008). Consequently, a single-copy gene in an ancestral angiosperm a million years ago could have expanded into a large gene family in recent species by WGD (Semon and Wolfe, 2007). TD is another important way for gene expansion. Genes expanded by TD are always distributed together as a cluster in chromosomes.

Whole genome duplication differs from TD in that WGD increases the dosage of all genes simultaneously and creates duplicate, potentially redundant, copies of all the genes within a genome. As reported previously, gene families expanded by WGD could maintain proper balance in the biological network or cascade (Freeling and Thomas, 2006). After WGD and following gene fractionation, the number of genes responding to abiotic and biotic stresses and with membrane protein functions was increased (Rizzon et al., 2006). TD is the most studied mechanism for the expansion of some gene families, such as genes that respond to environmental factors. In plants that cannot escape from stresses, who must endure turbulently changing environments and prevent themselves from being wounded (Freeling, 2009), the genes related to stress defense need to expand to resist environmental stimulation. It has been reported that TD expanded genes were more closely associated with stress-related functions than the non-TD expanded genes (Parniske et al., 1997; Michelmore and Meyers, 1998; Lucht et al., 2002; Kovalchuk et al., 2003; Leister, 2004; Shiu et al., 2004; Maere et al., 2005; Mondragon-Palomino and Gaut, 2005; Rizzon et al., 2006). Thus, the amplification of stress response genes by TD is regarded as a mechanism for protecting plants from harmful stresses. Tandem genes can be divided into classes based on gene dosage. The low-TD class appears to be represented by highly conserved, housekeeping, or key regulatory gene families, such as the transcription factor families and the proteasome 20S subunit family, while the medium- and high-TD classes are represented by gene families involved in responses to abiotic and biotic stimulus, such as pathogen defenses like NBS-LRR, or diverse enzymatic functions (Cannon et al., 2004). In comparison to WGD, TD occurred much more frequently and was responsible for the adaptive evolution of plants to rapidly changing environments (Hanada et al., 2008).

Genes are amplified through WGD and TD in a biased manner. According to gene dosage constraints (Edger and Pires, 2009), there was a functional bias in genes retained after WGD and TD. Gene families that need to maintain proper balance in the biological network or cascade were over-retained after WGD (Freeling and Thomas, 2006). In *Brassica rapa*, the genes expanded via whole genome triplication (WGT) tend to be in functional categories such as transcriptional regulation, ribosomes, response to abiotic or biotic stimuli, response to hormonal stimuli, cell organization, and transporter functions. In contrast, TD is responsible for the expansion of genes in categories such as response to environmental stimuli, defense response, various transport functions, and metabolism. The relationship between WGT and TD shows positive or negative correlations in the expansion of different gene families. Recent studies (Hanada et al., 2008) indicated that genes involved in biotic stress responses, such as the Receptor-Like kinase family, were over-retained by both polyploidy (WGD) and local duplication (TD) in *Arabidopsis thaliana* lineages, demonstrating positive correlation. However, some gene categories, such as “transcription factors” and “ribosomal proteins,” were over-retained post-WGD (Edger and Pires, 2009; Freeling, 2009), but under-retained post-TD (Freeling and Thomas, 2006), which shows the negative relationship. The Gene Balance Hypothesis can explain the reciprocal pattern (negative relationship) between WGD and TD. However, the impact of WGD on the evolution of tandem genes is still unknown.

**Figure A1:**
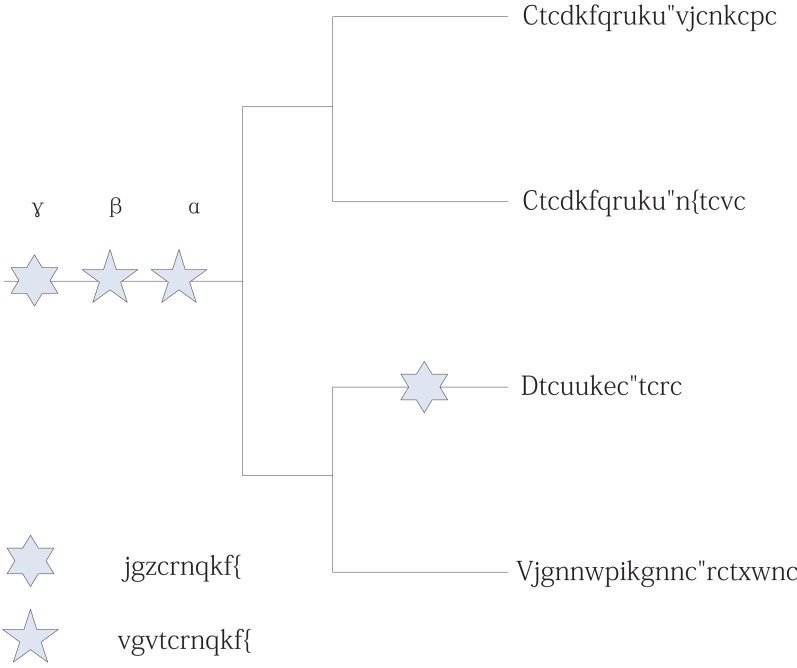
**The phylogenetic tree as above comes from website (http://www.phytozome.net/)**.

In this work, we studied the impact of WGT on tandem gene evolution in the recently sequenced genome of *B. rapa*, which is one of the most important vegetable crops. The annotation of *B. rapa* whole genome sequences provides the opportunity for the study of gene expansion after WGT (Wang et al., 2011), and the *B. rapa* genome serves as a model system to study the impact of WGT on tandem gene evolution. *A. thaliana*, *Arabidopsis lyrata*, *Thellungiella parvula*, and *B. rapa* belong to the family Brassicaceae and have a close relationship in the phylogenetic tree (Figure A1 in Appendix). They have undergone recurring WGD and TD in their evolutionary history. *B. rapa* underwent an additional genome triplication compared with the genomes of *A. thaliana*, *A. lyrata*, and *T. parvula*. The four species share a common ancestor from Brassicaceae, and the lineages of these three plants, *A. thaliana* (The Arabidopsis Genome Initiative, 2000), *A. lyrata* (Hu et al., 2011), and *T. parvula* (Dassanayake et al., 2011), can be regarded as controls for the pre-WGT lineages of *B. rapa*. Here, *A. thaliana*, *A. lyrata*, and *T. parvula* were used as out-groups for the investigation of the impact of WGT on the evolution of tandem genes in *B. rapa*.

## Results

### Tandem duplication in *A. thaliana*, *A. lyrata*, *T. parvula*, and *B. rapa*, and the shared syntenic tandem arrays among them

**Figure 1:**
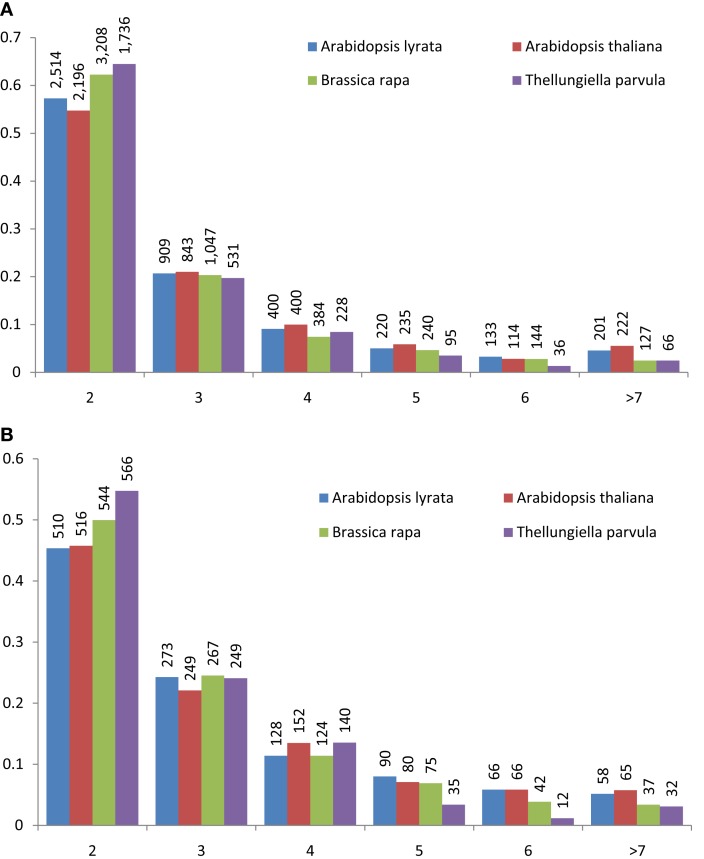
**Distribution of tandemly repeated gene arrays in the *Arabidopsis thaliana*, *Arabidopsis lyrata*, *Brassicarapa*, and *Thellungiella parvula* genomes**. The number of genes in each tandem array mostly ranged from 2 to 6; data from tandem arrays with more than seven genes was combined. Tandemly repeated gene arrays were identified using the BLASTP program with a threshold of *E* < 10^−20^. One unrelated gene among cluster members was tolerated. In both **(A)** and **(B)**, the frequency of tandem gene number is shown on the vertical axis, and the number of tandemly duplicated genes in the arrays is shown below the horizontal axis. The histogram shows the number of clusters in the genome containing two to *n* similar gene units in tandem. **(A)** The distribution of gene number in all tandem arrays of *A. thaliana*, *A. lyrata*, *B. rapa*, and *T. parvula*. **(B)** The distribution of gene number in the shared syntenic tandem arrays among *A. thaliana*, *A. lyrata*, *B. rapa*, and *T. parvula*.

**Figure 2:**
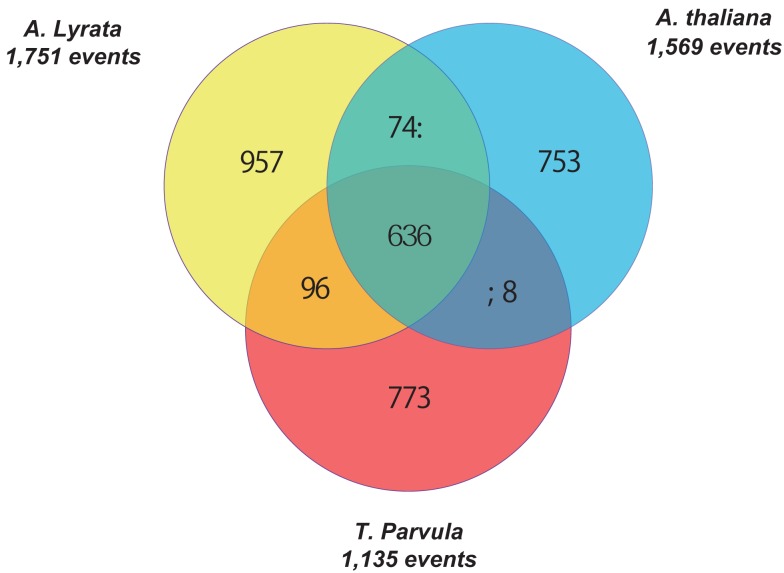
**Venn diagram showing unique and shared tandem arrays between and among the three dicotyledonous species (*Arabidopsis thaliana*, *Arabidopsis* lyrata, and Thellungiella parvula)**.

Tandem gene arrays contain homologous duplicates that are near to each other. Here, tandemly arrayed genes were defined as a list of paralogous genes (≥2) with sequence homology satisfying the BLASTP *E*-value < 10^−20^, and they should not contain more than one intervening gene among them (The Arabidopsis Genome Initiative, 2000). With the above rules, we detected 2,137, 1,569, 1,751, and 1,135 tandem gene arrays in *B. rapa*, *A. thaliana*, *A. lyrata*, and *T. parvula*, respectively. The distribution of the number of paralogous genes in each tandem array is shown in Figure 1A. Tandem arrays with two genes were predominant. The distribution of gene numbers in tandem arrays was not significantly different among the four species. Among these tandem gene arrays, 414 were syntenic tandem arrays shared among *A. thaliana*, *A. lyrata*, and *T. parvula* (Figure 2; Table 1 in Supplementary Material). The distribution of gene numbers in shared tandem arrays is shown in Figure 1B. It was similar to the distribution of all tandemly duplicated genes in Figure 1A. These shared tandem arrays should have originated from a common ancestor and been retained by all three species. This set of shared tandem gene arrays was used as the set of presumed ancestral tandem arrays, to investigate their evolution in *B. rapa* after WGT.

The number of genes that the 414 conserved tandem arrays contain in the three non-WGT species is 1,093 in *A. thaliana*, 1,090 in *A. lyrata*, and 998 in *T. parvula*. Of 414 tandem arrays, 400 had at least one syntenic ortholog in the genome of *B. rapa* (Table 1 in Supplementary Material). Among these 400 tandem arrays, 294 were syntenic to at least one tandem array in the three sub-genomes of *B. rapa*. Of 294 tandem arrays, 178 only exist in one sub-genome while 100 tandem arrays exist in two sub-genomes. However, only 16 tandem arrays exist in all three sub-genomes (LF, MF1, and MF2) simultaneously. Because *B. rapa* experienced an extra WGT, some tandem arrays had two or three copies in *B. rapa* in total, there were 426 tandem arrays in *B. rapa* corresponding to these 294 shared tandem arrays (Table 1 in Supplementary Material). The gene number in the 426 tandem arrays of *B. rapa* is 1,096. After the post-WGT fractionation, 426 tandem arrays maintained TDs, 418 tandem arrays were reduced to one copy, and 398 tandem arrays disappeared. These distributions are shown in Table 1.

**Table 1:** **The distribution of gene numbers in tandem arrays from three sub-genomes of *Brassica rapa* after whole genome triplication (WGT)**.

	LF	MF1	MF2	Total
0 gene	88	137	173	398
1 gene	141	144	133	418
>1 genes	185	133	108	426
Total	414	414	414	1,242

*“0 gene” refers to the genes in the tandem arrays of the B. rapa’s ancestor that were all lost in the sub-genome of B. rapa*.

*“1 gene” refers to the genes in the tandem arrays of the B. rapa’s ancestor that were fractionated to a single gene in the sub-genome of B. rapa*.

*“>1 genes” refers to the tandem arrays of the B. rapa’s ancestor that were maintained in the sub-genome of B. rapa*.

### Impact of WGT on shared tandem gene arrays in *B. rapa*

Compared with *A. thaliana*, *A. lyrata*, and *T. parvula*, *B. rapa* experienced an additional genome triplication, thus the ratio of syntenic genes between *B. rapa* and the three species should be 3:1. However, the ratio was much smaller for genes that were fractionized following WGT (Woodhouse et al., 2010). If WGT has no impact on TD then the ratio of syntenic tandem arrays between *B. rapa* and the other three species should be consistent with the ratio of syntenic non-tandem genes between them.

We identified 15,791 shared syntenic genes among *A. thaliana*, *A. lyrata*, and *T. parvula*, of which 13,915 genes had syntenic orthologs in *B. rapa* (Table 1 in Supplementary Material). Due to the WGT and subsequent gene fractionation, the total number of syntenic genes in *B. rapa* was 22,630 corresponding to the 13,915 syntenic orthologs (Cheng et al., 2012a; Table 1 in Supplementary Material). These 22,630 genes can be regarded as the genes retained in *B. rapa* after its genome triplication. If the WGT has no specific impact on the evolution or the loss of tandem gene arrays, the ratio of tandem arrays retained in *B. rapa* to the other three species should be in accordance with the ratio observed for all non-tandem genes. However, they were significantly different from each other (*P*-value=1.30e-06, Table 2). We also performed individual tests between *B. rapa* and *A. thaliana*, *B. rapa* and *A. lyrata*, as well as *B. rapa* and *T. pravula* (Table 3). The *P*-values were 3.47e-03, 1.35e-03, and 1.85e-17, respectively. These results indicated that tandem gene arrays in *B. rapa* significantly decreased after WGT compared with the non-WGT species.

**Table 2:** **Statistical test between the number of syntenic tandem arrays and all syntenic genes among *Arabidopsis thaliana*, *Arabidopsis lyrata*, and *Thellungiella parvula*, and in *Brassicarapa***.

	Number of shared tandem arrays	Number of syntenic genes	*P*-value^c^
^a^*At-Aly-Tp*	414	15,791	1.303e-06
^b^Syntenic in *B. rapa*	426	22,630	

*^a^The number of shared tandem arrays and syntenic genes among A. thaliana, A. lyrata, and T. parvular*.

*^b^The number of shared tandem arrays and syntenic genes in B. rapa*.

*^c^Fisher’s exact test*.

**Table 3:** **Fisher’s exact test between the number of shared syntenic tandem arrays and all syntenic non-tandem genes in *Arabidopsis thaliana, Arabidopsis lyrata, Thellungiella parvula*, and *Brassica rapa*, respectively**.

	Number of shared tandem arrays	Number of syntenic gene	*P*-value^a^
*A. thaliana*	658	18,388	3.47e-03
*B. rapa*	917	29,538	
*A. lyrata*	603	18,125	1.35e-03
*B. rapa*	854	30,250	
*T. parvula*	524	17,303	1.85e-17
*B. rapa*	524	29,433	

*^a^Fisher’s exact test*.

We further looked into the loss of tandem arrays in each of the three sub-genomes of *B. rapa*: the least fractionated genome (LF), the medium fractionated genome (MF1), and the most fractionated genome (MF2; Wang et al., 2011; Cheng et al., 2012b). The *P*-values of Fisher’s exact test with LF, MF1, and MF2 were 2.08e-05, 8.21e-04, and 3.29e-03, respectively (Table 4), indicating that tandem arrays in LF, MF1, and MF2 sub-genomes were significantly decreased in *B. rapa*. Furthermore, paired *t*-tests on gene numbers for each shared tandem array between the *B. rapa* sub-genomes and the other three species showed that the gene numbers in tandem arrays of *B. rapa* were significantly less than in *A. thaliana*, *A. lyrata*, and *T. parvula* (Table 2 in Supplementary Material). On the whole, the number of shared tandem arrays and the number of genes in tandem arrays were significantly decreased in *B. rapa* after the WGT.

**Table 4:** **Statistical test between the number of tandem arrays and the number of non-tandem genes in the three species, *Arabidopsis thaliana, Arabidopsis lyrata*, and *Thellungiella parvula*, and *Brassica rapa*’s three sub-genomes**.

	Number of tandem arrays	Number of gene	*P*-value^a^
LF of *B. rapa*	185	10,145	2.08e-05
*At-Aly-Tp*	414	15,791	
MF1 of *B. rapa*	133	6,950	8.21e-04
*At-Aly-Tp*	414	15,791	
MF2 of *B. rapa*	108	5,535	3.29e-03
*At-Aly-Tp*	414	15,791	

*^a^Fisher’s exact test*.

### The evolution of tandem genes between species without the extra WGT

To verify the impact of WGT on the loss of tandem arrays in *B. rapa*, we selected *A. lyrata*, which has not experienced an extra WGT, and performed the same tests as on *B. rapa*. If the tandem arrays in *A. lyrata* decreased significantly, as was observed in *B. rapa*, then the loss of tandem genes in *B. rapa* would not be the impact of the WGT but a general process in different species. For the 391 syntenic tandem arrays shared among *A. thaliana*, *B. rapa*, and *T. parvula*, 336 can be found in *A. lyrata* (Table 3 in Supplementary Material). Meanwhile, for the 16,063 shared syntenic genes among *A. thaliana*, *B. rapa*, and *T. parvula*, 15,327 had syntenic orthologs in *A. lyrata* (Table 3 in Supplementary Material). Fisher’s exact test for the loss of the tandem arrays and non-tandem genes in *A. lyrata* gave a *P*-value of 0.08733 (Table 5). The tandem arrays in *A. lyrata* did not significantly decrease. This result demonstrated that WGT accelerated the loss of tandem arrays in *B. rapa*.

**Table 5:** **Statistical test between the number of shared syntenic tandem arrays and all syntenic non-tandem genes in *Arabidopsis thaliana, Brassica rapa*, and *Thellungiella parvula*, and *Arabidopsis lyrata***.

	Number of shared tandem arrays	Number of syntenic gene	*P*-value^b^
^a^*At-Bra-Tp*	391	16,063	0.088
*A. lyrata*	336	15,327	

*^a^Tandem arrays with a shared syntenic relationship among A. thaliana, B. rapa, and T. parvula*.

*^b^Fisher’s exact test*.

## Discussion

*Brassica rapa* experienced both a WGT and local duplications. Since its divergence from *A. thaliana*, *A. lyrata*, and *T. parvula*, *B. rapa* has undergone genome triplication, extensive gene fractionation, and genomic reshuffling that eroded its resemblance to ancestral Brassicales (Wang et al., 2011). The modes of WGD and TD in gene expansion have a reciprocal relationship. Furthermore, the WGD should have had an impact on tandem gene evolution. It is clear from our results that the shared tandem arrays among *A. thaliana*, *A. lyrata*, and *T. parvula* decreased significantly in *B. rapa* compared with the syntenic non-tandem genes. However, the shared tandem arrays among *A. thaliana*, *B. rapa*, and *T. parvula* did not significantly decrease in *A. lyrata*.

Previous reports showed that tandem duplicated genes tend to be involved in responses to stress or environmental stimuli (Parniske et al., 1997; Michelmore and Meyers, 1998; Lucht et al., 2002; Kovalchuk et al., 2003; Leister, 2004; Shiu et al., 2004; Maere et al., 2005; Mondragon-Palomino and Gaut, 2005; Rizzon et al., 2006) in plants and their cells. The need for stress endurance makes plants rich in genes associated with environmental factor responses. WGD expanded all genes in *B. rapa* simultaneously, including genes that respond to environmental factors. For example, one gene that defends against abiotic or biotic stimuli is increased to three genes through WGT in *B. rapa*. Though genes were rapidly fractionized and lost following WGD, genes that respond to environmental adaptability and hormones were still over-retained after WGT (Table 4 in Supplementary Material). There were many stress resistance genes generated from WGT in *B. rapa*, so it need not amplify these genes by TD. Additionally, redundant genes in response to abiotic and biotic stimuli would be lost during the evolution of tandem genes after their WGT expansion.

It has been characterized that tandem genes experience a rapid birth-and-death evolution (Nei and Rooney, 2005). Rapid birth-and-death evolution has occurred in many gene families that have tandem duplicates, such as plant disease resistance genes (Parniske et al., 1997; Michelmore and Meyers, 1998). With this feature, tandem genes would disappear in original positions and appear or expand in other genomic regions in *B. rapa*. That would also lead to a decrease in syntenic tandem arrays among *B. rapa* and other species.

## Conclusion

The evolution of tandemly duplicated genes in *B. rapa* has been affected by the WGT event. Following WGT, the triplicated tandem genes in *B. rapa* were largely lost. The ratio of lost tandem arrays is significantly larger than the ratio of lost non-tandem genes. All ancestral tandem arrays were triplicated by WGT in *B. rapa*. Genes in these triplicated tandem arrays then became functionally redundant and were prone to be lost in *B. rapa*, both in the number of tandem arrays and in the number of genes in each tandem.

## Materials and Methods

### Data sources

Genomic data for the four plant species (*A. thaliana*, *A*. *lyrata*, *T*. *parvula*, and *B. rapa*) were obtained from the databases of The Arabidopsis Information Resource (TAIR)^1^, the Joint Genome Institute^2^, the *T. parvula* genome sequencing group, and the *Brassica* database (BRAD^3^; Cheng et al., 2011).

### Tandem array identification

Tandem gene arrays were defined as homologous gene clusters with no more than one intervening gene located among them and sequence homology of the homologous genes in the array should satisfy BLASTP *E*-value<10^−20^.

### Syntenic gene identification

Syntenic genes between *A. thaliana* and *B. rapa*, *A. thaliana* and *A. lyrata*, and *A. thaliana* and *T. parvula* were identified using SynOrths (Cheng et al., 2012a). We took one gene from each tandem array as a representative to determine the syntenic relationships among the four species.

## Conflict of Interest Statement

The authors declare that the research was conducted in the absence of any commercial or financial relationships that could be construed as a potential conflict of interest.

## Supplementary Material

The Supplementary Material for this article can be found online at http://www.frontiersin.org/Plant_Genetics_and_Genomics/10.3389/fpls.2012.00261/abstract

## Footnotes

^1^www.arabidopsis.org

^2^http://genome.jgi-psf.org/Araly1/Araly1.home.html

^3^http://brassicadb.org/brad/
